# Surfactant‐Free Continuous‐Flow Synthesis of Cu_2_O Crystals with Diverse Facets and Sizes

**DOI:** 10.1002/smtd.202501927

**Published:** 2025-11-29

**Authors:** Chunli Han, Akira Yoko, Ardiansyah Taufik, Satoshi Ohara, Tadafumi Adschiri

**Affiliations:** ^1^ World Premier International Research Center Initiative‐Advanced Institute for Materials Research (WPI‐AIMR) Tohoku University Sendai 980‐8577 Japan; ^2^ International Center for Synchrotron Radiation Innovation Smart Tohoku University Sendai 980‐8572 Japan; ^3^ New Industry Creation Hatchery Center Tohoku University Sendai 980‐8579 Japan

**Keywords:** continuous‐flow synthesis, Cu_2_O, kinetic control, nanocube, polyhedron

## Abstract

Cuprous oxide (Cu_2_O) has demonstrated great potential in photochemical, electrochemical, and organic catalysis. Developing surfactant‐free and scalable synthesis methods is essential for its real application. Conventional batch methods often suffer from inconsistent product quality and limited scalability. In this work, an efficient continuous microflow synthesis system is developed, and the design principles of the flow synthesis system are systematically elucidated. A kinetic control strategy on the millisecond‐to‐second timescale is proposed to precisely regulate intermediate size and dynamic structural evolution without using surfactants, thereby adjusting particle size and exposed crystal facets, which transformed the conventional thermodynamic control paradigm. Specifically, varying the interval time (0.02→6 s) between precipitant (sodium hydroxide, NaOH) and reductant (ascorbic acid, AA) addition significantly altered nanocube size (75→196 nm), while reversing the feeding sequence (AA before NaOH) led to much smaller nanocubes (76→14 nm) due to changes in the microenvironments for particle formation. Moreover, Cu_2_O polyhedrons exhibited a greater number of exposed facets at shorter residence times, indicating a non‐equilibrium state from the thermodynamic perspective. It is expected that such a continuous microflow synthesis system can be directly integrated with downstream catalytic processes to fully exploit the activity of Cu_2_O.

## Graphical abstract

Precise and scalable synthesis is crucial to the practical applications of functional materials. In this work, we demonstrate the significant influence of precise process control on the formation of Cu_2_O crystals based on a continuous microflow synthesis system. The millisecond‐to‐second kinetic control is highlighted, which can substantially modify the microenvironment and intermediate states, thereby dictating the size and exposed facets.

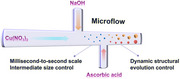

## 1 Introduction

Cuprous oxide (Cu_2_O) is a highly active and efficient material widely applied in electrochemical,^[^ ^1^, ^2^, ^3^ ^]^ photochemical,^[^ ^4^, ^5^, ^6^ ^]^ photoelectrochemical,^[^ ^7^, ^8^, ^9^ ^]^ photothermal catalysis,^[^ ^10^, ^11^ ^]^ and sensing applications.^[^ ^12^ ^]^ Its performance is strongly influenced by morphology, particle size, and surface property. The synthesis methods of Cu_2_O nanocubes, polyhedrons, and spheres in batch reactors have been well established,^[^ ^13^, ^14^, ^15^, ^16^, ^17^ ^]^ typically employing inorganic copper salts or copper acetate as precursors, NaOH as the precipitant, and ascorbic acid (AA), glucose, NH_2_OH·HCl, or NaBH_4_ as the reductant. Surfactants, such as polyvinylpyrrolidone (PVP), sodium dodecyl sulfate (SDS), or citric acid, are often introduced to manipulate thermodynamically stable states, enabling further control over particle size and exposed facets.^[^ ^18^ ^]^

However, for catalytic applications, clean surfaces are crucial to ensure optimal interaction between reactants and the catalyst surface. Therefore, developing surfactant‐free methods for precise control of morphology and size is significant for enhancing downstream application performance. Another key limitation of conventional batch methods is their inherently low production yield under typical synthesis conditions, and the scale‐up to industrial production is hindered by common drawbacks such as relatively poor mixing efficiency and batch‐to‐batch variability. Advancing controllable and scalable synthesis techniques is therefore critical for the future industrial implementation of Cu_2_O nanocrystals.

Continuous‐flow technology has been widely used in material synthesis, spanning conditions from ambient to supercritical hydrothermal states, as reviewed in recent literature.^[^ ^19^, ^20^, ^21^, ^22^, ^23^, ^24^, ^25^, ^26^, ^27^ ^]^ Its unique advantages in enhanced heat and mass transfer, precise process control, and straightforward scalability make it an advanced platform not only for precise and scalable material synthesis but also for mechanistic investigations and the discovery of novel materials. For example, Völkl et al. developed a gram‐scale continuous flow system for the reproducible and tunable synthesis of silver‐on‐silica patchy nanoparticles for plasmonic applications.^[^ ^28^ ^]^ Yoko et al. successfully synthesized ultrasmall (<5 nm) CeO_2_ nanoparticles using a flow reactor, which exhibited disordered atomic configurations and unusual electron localization.^[^ ^29^ ^]^ Schmitt et al. reported a continuous stirred tank reactor for the atom‐precise synthesis of platinum clusters.^[^ ^30^ ^]^ Moreover, thermodynamically non‐equilibrium metal oxide nanoparticles were captured by flow reactors, exhibiting superior lattice oxygen storage and release capacity.^[^ ^31^, ^32^, ^33^ ^]^ Continuous‐flow platforms have also been increasingly utilized for highly efficient material discovery by integrating with in‐situ characterization, autonomous robotic experimentation, and AI‐driven optimization strategies.^[^ ^34^, ^35^, ^36^ ^]^ The potential of continuous‐flow synthesis is expected to be further explored and exploited.

Currently, reports on the flow synthesis of Cu_2_O are very limited. Jun et al. demonstrated a microfluidic‐assisted synthesis of hierarchical Cu_2_O nanocrystals, where the mixing efficiency between the reaction solution and reductant significantly influenced crystal growth kinetics and final morphology.^[^ ^37^ ^]^ Ebri et al. developed a high‐temperature (200–230 °C) flow synthesis system to overcome the deactivation limitations of Cu_2_O photocatalysts, achieving tunable Cu_2_O/Cu^0^ ratios by adjusting residence time.^[^ ^38^ ^]^ Despite these advances, continuous‐flow synthesis strategies enabling precise control over Cu_2_O nanocrystal size and exposed facets, particularly for ultrasmall nanocubes and multi‐faceted polyhedrons, remain unexplored.

In this work, a continuous‐flow system for synthesizing Cu_2_O nanocrystals was developed, with copper nitrate hydrate (Cu(NO_3_)_2_·3H_2_O), sodium hydroxide (NaOH), and ascorbic acid (AA) as reactants. The design principles, including Reynolds number (*Re*) and stage‐specific residence times, were systematically elucidated. Key synthesis parameters (e.g., concentrations of NaOH, AA, and Cu precursor, reaction temperature, and AA/NaOH feeding sequence) were systematically investigated, along with strategies for increasing productivity. Morphology and size were precisely tuned by kinetic control methods without surfactants, successfully yielding Cu_2_O nanocubes, nanospheres with porous and smooth surfaces, polyhedrons with different facet numbers.

## 2 Experimental Section

### 2.1 Materials

Copper nitrate trihydrate (Cu(NO_3_)_2_·3H_2_O, 100%), L(+)‐ascorbic acid (C_6_H_8_O_6_, 99.6%), sodium hydroxide (NaOH, 97%), and ethanol (C_2_H_5_OH, 99.5%) were purchased from FUJIFILM Wako Pure Chemical Corporation (Japan). All chemicals were used as received without any further purification. Deionized water was used for all the experiments.

### 2.2 Continuous‐Flow Synthesis of Cu_2_O Crystals

**Figure 1:**
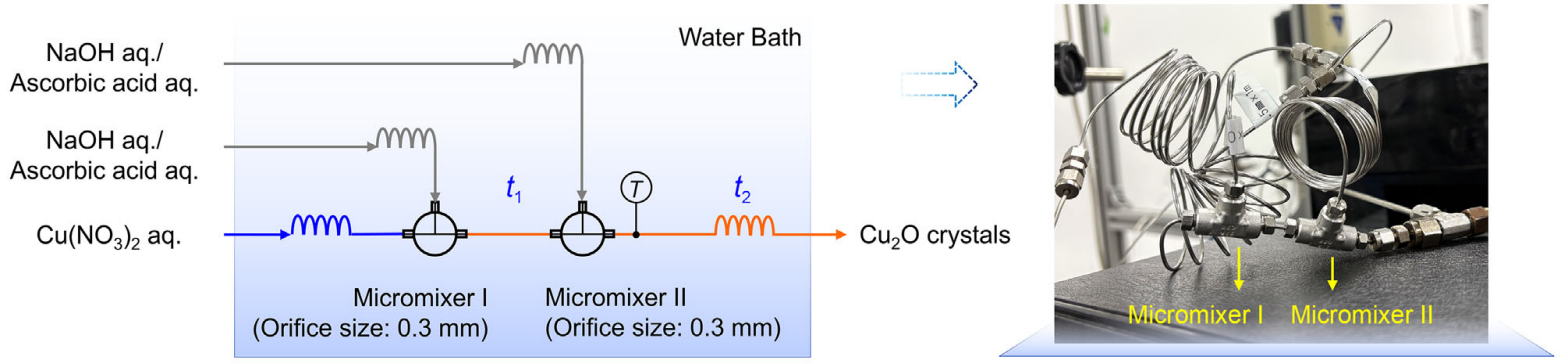
Schematic and photograph of the Cu_2_O continuous‐flow synthesis system.

**Figure** **1** illustrates the schematic of the continuous‐flow synthesis system for Cu_2_O crystals. The entire setup was immersed in a water bath to maintain a constant reaction temperature in the range of 10–70 °C. Prior to mixing, each solution first flowed through a coiled stainless‐steel tube (1/16″ outer diameter, 0.5 mm inner diameter, 1 m length) to reach the target temperature. Typically, a Cu(NO_3_)_2_ solution was first mixed with a NaOH solution using a micromixer (Swagelok, SS‐1F0‐3GC, orifice size: 0.3 mm). Subsequently, an AA solution was introduced via a second micromixer of the same specification. The actual reaction temperature was monitored using a thermocouple positioned downstream of the second micromixer. Particle growth then occurred in a tubular reactor. The time intervals between NaOH and AA feeding, particle growth in the tubular reactor, and aging in the collection vessel were defined as *t*_1_, *t*_2_, and *t*_3_, respectively. The collected samples were centrifuged at 10 000 rpm for 60 min, then washed twice with ethanol and water (volume ratio 1:1), and dried in a vacuum oven at 60 °C for 3 h. To evaluate the conversion rate of the Cu precursor, the supernatant was further centrifuged at 10 000 rpm for 60 min to remove the trace nanoparticles. In addition, the effect of the feeding sequence of NaOH and AA was investigated.

### 2.3 Characterization

The morphologies of Cu_2_O crystals were examined using transmission electron microscopy (TEM, HITACHI H‐7650, Japan) operated at an accelerating voltage of 100 kV, and a field‐emission scanning electron microscope (FE‐SEM, JEOL JSM‐7800F, Japan) operated at an accelerating voltage of 15 kV. The average particle size was obtained by measuring 200 particles using Nano Measurer 1.2 (Fudan Univ., China). The diffraction patterns of Cu_2_O crystals were collected using X‐ray powder diffraction (XRD, Rigaku SmartLab 9MTP, Japan) with a Cu Kα radiation source (*λ *= 1.5418 Å) at 3° min^−1^ at a 2*θ* scanning range of 20°–80°. The conversion rate of the Cu precursor was analyzed using inductively coupled plasma‐atomic emission spectrometry (ICP‐AES).

## 3 Results and Discussion

### 3.1 Design of the Microflow Continuous Synthesis System

#### 3.1.1 Reynolds Number

**Figure 2:**
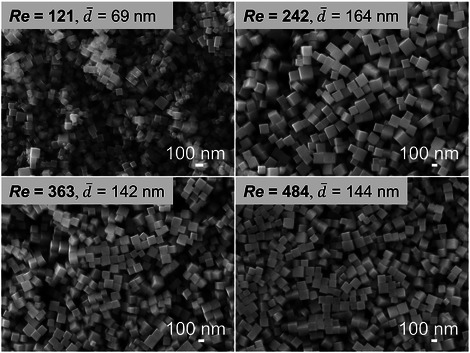
SEM images of Cu_2_O nanocubes synthesized under different Reynolds numbers (*Re*). Here, *Re* refers to the Reynolds number in the tubular reactor between micromixer I and II. Other synthesis conditions: *T* = 30 °C, After mixing: *C*_Cu_ = 0.002 m, *C*_AA_ = 0.01 m, *C*_NaOH_ = 0.02 m, *t*_1_ = 0.5 s, *t*_2_ = 26 s, *t*_3_ = 20 min.

The Reynolds number is a critical parameter in continuous‐flow particle synthesis systems, as it directly influences mixing efficiency, back‐mixing extent, and particle deposition within the tubular reactor. In this study, an appropriate *Re* for the designed tubular reactor was first determined by proportionally adjusting the flow rates of reactant solutions. As shown in **Figure** **2**, a broad size distribution of Cu_2_O nanocubes was observed at a low *Re* of 121. In contrast, when *Re* exceeded 242, the particle size distribution became markedly narrower, which could be attributed to less back‐mixing and particle deposition. Based on these observations, a *Re* of 363 was selected as optimal for synthesizing Cu_2_O nanocubes. This corresponded to a flow rate of 18 mL min^−1^ for Cu(NO_3_)_2_ solution (*F*_Cu_), 6 mL min^−1^ for NaOH solution (*F*_NaOH_), and 3 mL min^−1^ for AA solution (*F*_AA_). Under these conditions, the *Re* in the mixing zone of the first micromixer can be calculated as follows:

(1) $$Re=\frac{d\rho u}{\mu }$$

where *d* is the characteristic size of the micromixier (0.3 mm), *ρ* and *µ* are the density and viscosity of water, respectively, and *u* is the fluid velocity at the mixing point. The calculated *Re* is 2120. Then, the micromixing efficiency can be evaluated based on Kolmogorov's theory.^[^ ^39^, ^40^ ^]^ The Kolmogorov length scale *η* is defined as

(2) $$\eta ={\left(\frac{\nu^{3}}{\varepsilon }\right)}^{1/4}$$

where *ν* is kinematic viscosity, and *ε* is energy dissipation rate. *ε* can be estimated by

(3) $$\varepsilon ∼\frac{u^{3}}{d}$$

where *u* is the average flow velocity at the mixing point, and *d* is the characteristic size of the micromixer.

Then, the micromixing time τ_*m*_ can be approximated as

(4) $$\tau_{m}\approx \tau_{diffusion}∼\frac{\eta^{2}}{D}$$

where *D* is the molecular diffusivity.

The calculated micromixing time *τ*_*m*_ is 4.5 × 10^−5^ s, i.e., 0.045 ms at *Re* = 2120, which is much shorter than the subsequent reaction times *t*_1_ and *t*_2_. Therefore, the morphology and size of the as‐synthesized Cu_2_O were mainly affected by the reactions, nucleation/growth processes, and fluid flow rather than by the mixing efficiency.

#### 3.1.2 Residence Time

In batch processes, process control is relatively rough, and the effects of the interval time between NaOH and AA addition and the reaction time after AA introduction have been rarely studied, presenting a key challenge in the rational design of a continuous‐flow synthesis system. To address this, we investigated the effects of residence times following NaOH and AA additions, ranging from milliseconds to second scale.

**Figure 3:**
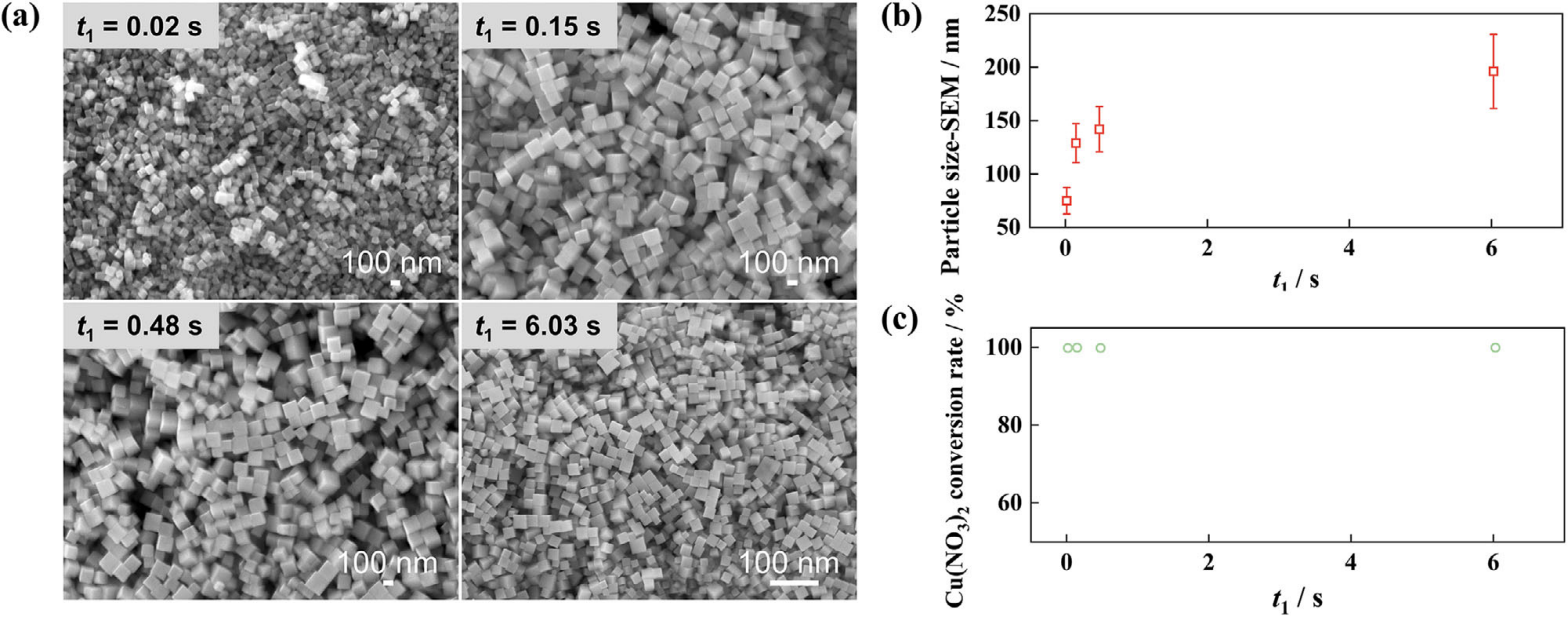
Effect of interval time between NaOH and AA addition on the size of Cu_2_O crystals. a, b) SEM images and average particle sizes of Cu_2_O crystals synthesized with varying *t*_1_. The error bar in b) represents the standard deviation of particle size in each sample. c) Conversion rates of the Cu precursor at different *t*_1_. Other synthesis conditions: *T* = 30 °C, After mixing: *C*_Cu_ = 0.002 m, *C*_AA_ = 0.01 m, *C*_NaOH_ = 0.02 m, *t*_2_ = 26 s, *t*_3_ = 20 min.

**Figure 4:**
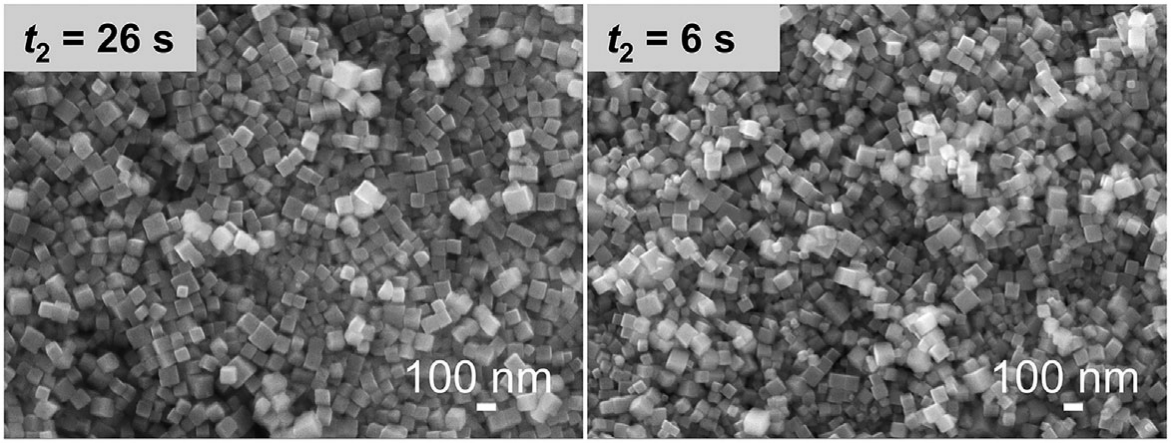
Effect of residence time after AA addition (*t*_2_) on the size distribution of Cu_2_O crystals. Other synthesis conditions: *T* = 30 °C, After mixing: *C*_Cu_ = 0.002 m, *C*_AA_ = 0.01 m, *C*_NaOH_ = 0.02 m, *t*_1_ = 0.02 s, *t*_3_ = 0 min.

**Figure** **3**a presents SEM images of Cu_2_O nanocubes synthesized with varying interval times (*t*_1_) between NaOH and AA addition. All samples exhibited narrow size distributions. As *t*_1_ increased from 0.02 to 6.03 s, the average nanocube size increased from 75 to 196 nm (Figure 3b), while the conversion rates of the Cu precursor all exceeded 99.8% at different *t*_1_ (Figure 3c). This suggests that the state of the Cu(OH)_2_ intermediate plays a critical role in the subsequent Cu_2_O formation. A longer *t*_1_ possibly results in larger Cu(OH)_2_ particles, reducing the reduction rate, lowering the nucleation density, and allowing more Cu species to participate in crystal growth, thus yielding larger Cu_2_O nanocubes. This millisecond‐scale kinetic control of Cu_2_O particle size is reported here for the first time, which is unachievable in the batch reactor. Subsequently, the effect of residence time after AA addition (*t*_2_) was studied. As shown in **Figure** **4**, a shorter *t*_2_ led to a broader size distribution, indicating that the rapid growth stage was not finished in the tubular reactor with a residence time of 6 s. By extending *t*_2_, the plug flow in the tubular reactor can suppress back‐mixing and ensure uniform nanocube growth. Furthermore, the effect of aging time in the collection vessel (*t*_3_) was examined. As shown in Figure S1 (Supporting Information), for Cu_2_O nanocube synthesis, aging had a negligible influence on particle morphology and size, indicating that particle growth was completed within the flow reactor with a residence time of 26 s. These insights provide a basis for the rational design and control of the Cu_2_O continuous‐flow synthesis system.

### 3.2 Effect of Synthesis Parameters and Productivity Enhancement Strategy

#### 3.2.1 Synthesis Parameters

**Figure 5:**
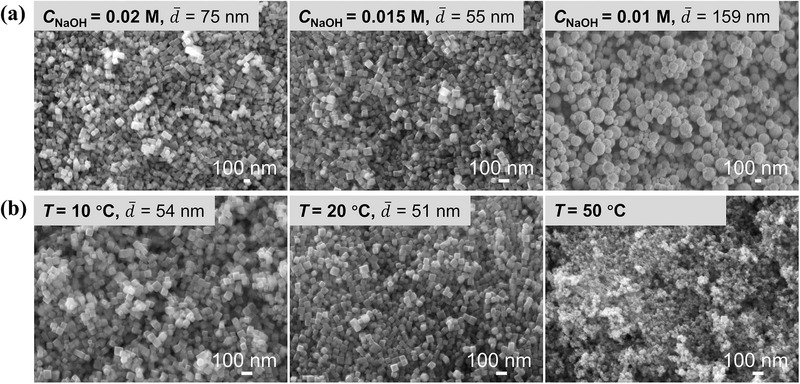
a) Effect of NaOH concentration on Cu_2_O crystals. Other synthesis conditions: *T* = 30 °C, After mixing: *C*_Cu_ = 0.002 m, *C*_AA_ = 0.01 m, *t*_1_ = 0.02 s, *t*_2_ = 26 s. b) Effect of temperature on Cu_2_O crystals. Other synthesis conditions: After mixing: *C*_Cu_ = 0.002 m, *C*_AA_ = 0.01 m, *C*_NaOH_ = 0.015 m, *t*_1_ = 0.02 s, *t*_2_ = 26 s.

To adjust the size and morphology of Cu_2_O nanocubes, key synthesis parameters were systematically investigated, including the concentrations of NaOH, AA, and Cu precursor, reaction temperature, and the feeding sequence of AA and NaOH. **Figure** **5**a shows SEM images of Cu_2_O nanoparticles synthesized with varying NaOH concentrations. Higher NaOH concentrations could promote the formation of Cu(OH)_4_^2^^−^ and enhance the reducibility of AA simultaneously, thereby accelerating Ostwald ripening and resulting in larger nanocubes. This trend is consistent with observations in batch synthesis. At a low NaOH concentration (0.01 m), porous nanospheres were formed because of the rapid aggregation of ultrasmall primary particles. When the NaOH concentration was fixed, increasing the AA concentration altered the morphology from nanocubes to nanospheres and irregular particles (Figure S2, Supporting Information), rather than reducing nanocube size. This may be attributed to the rapid reduction at high AA concentrations, leading to the instantaneous formation of a high density of nuclei. These primary particles tend to aggregate into spherical structures to minimize surface energy. Further increasing AA concentration significantly accelerated Ostwald ripening, resulting in large, irregular polyhedrons. Figure 5b displays SEM images of Cu_2_O synthesized at different temperatures. Between 10 and 30 °C, the morphology and size kept constant. However, at 50 °C, the as‐synthesized Cu_2_O became much smaller, ascribed to remarkably increased reduction rate and higher nucleation density at elevated temperature.

#### 3.2.2 Productivity Enhancement Strategy

**Figure 6:**
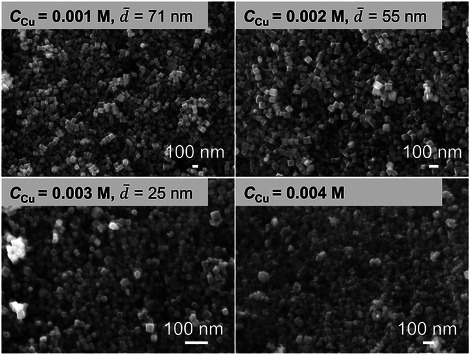
Effect of Cu precursor concentration on Cu_2_O crystals. Other synthesis conditions: *T* = 30 °C, After mixing: *C*_AA_ = 0.01 m, *C*_NaOH_ = 0.015 m, *t*_1_ = 0.02 s, *t*_2_ = 26 s.

Increasing precursor concentration is a common approach for improving productivity. However, the molar ratios among the Cu precursor, AA, and NaOH significantly influence Cu_2_O formation, as shown in **Figure** **6**. When NaOH and AA concentrations were kept constant, the increase in the Cu precursor concentration led to a reduction in nanocube size. This is attributed to a higher degree of supersaturation, which favors nucleation over growth. With a Cu precursor concentration of 0.004 m, irregular aggregates were observed. Subsequently, the concentrations of NaOH and AA were increased proportionally with the Cu precursor. Despite this adjustment, the Cu_2_O nanocube size continued to decrease with increasing Cu precursor concentration (Figure S3a–d, Supporting Information). With further fine‐tuning of the AA concentration, successful productivity enhancement with maintained nanocube size was achieved (Figure S3e, Supporting Information).

**Figure 7:**
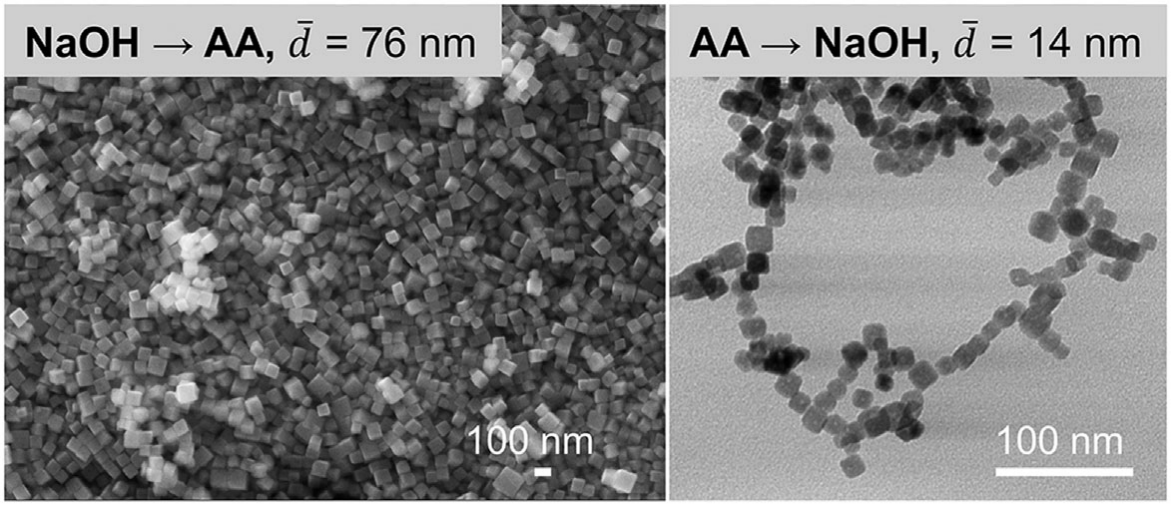
Effect of the feeding sequence of NaOH and AA on Cu_2_O crystals. a) First NaOH and then AA, b) first AA and then NaOH. Other synthesis conditions: *T* = 30 °C, After mixing: *C*_Cu_ = 0.002 m, *C*_AA_ = 0.01 m, *C*_NaOH_ = 0.02 m, *t*_1_ = 0.02 s, *t*_2_ = 26 s.

**Figure 8:**
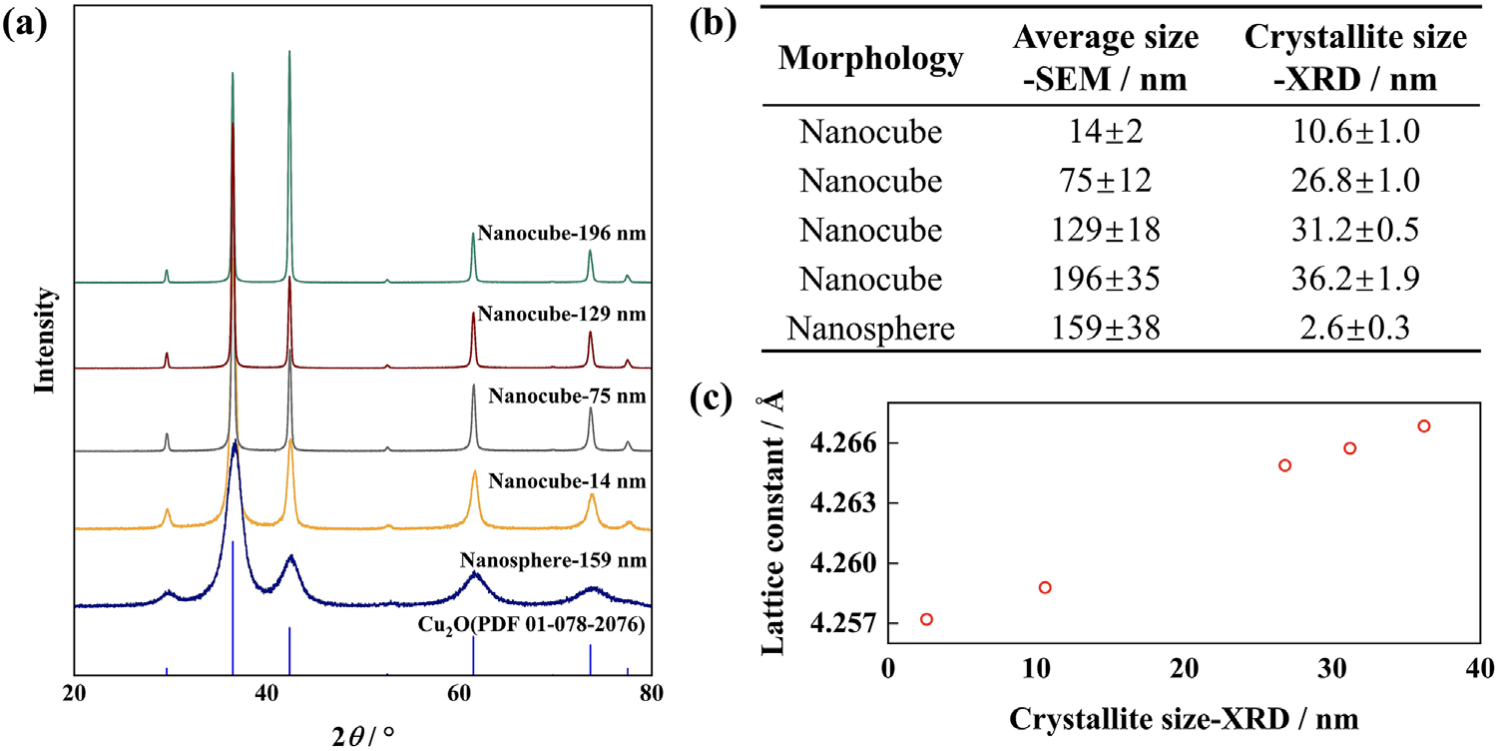
a) XRD patterns of representative Cu_2_O nanospheres and nanocubes. b) Average particle sizes and the corresponding crystallite sizes of the as‐synthesized Cu_2_O nanoparticles. c) Variation of the lattice constant of Cu_2_O nanocrystals with crystallite size.

By changing the concentrations of Cu precursor, AA, and NaOH, the smallest Cu_2_O nanocube size obtained was 25 nm (Figure 6). As discussed earlier, shortening the interval time between NaOH and AA addition effectively decreased the particle size. To further synthesize smaller Cu_2_O nanocubes, the feeding sequence of AA and NaOH was directly reversed, i.e., feeding AA before NaOH. Surprisingly, under otherwise identical conditions, the Cu_2_O nanocube size was reduced from 76 to 14 nm (**Figure** **7**). This dramatic size reduction could be attributed to the immediate reduction of Cu(OH)_2_ upon its formation in the AA‐rich environment, suppressing growth and promoting ultrafine crystallization. **Figure** **8**a shows the XRD patterns of representative nanospheres and nanocubes, with all diffraction peaks corresponding to Cu_2_O. Figure 8b,c exhibit the crystallite sizes and corresponding lattice constants of the as‐synthesized Cu_2_O nanocrystals. The lattice constant decreased with decreasing crystallite size, which is anomalous compared to the commonly observed lattice expansion in metal oxide nanoparticles.^[^ ^41^ ^]^ This behavior may originate from different environmental conditions and surface absorbates, which influence surface stress and thereby induce either lattice expansion or contraction. These variations in crystallite size and lattice constant are expected to result in distinct physicochemical properties.

### 3.3 Continuous‐Flow Synthesis of Cu_2_O Polyhedrons

**Figure 9:**
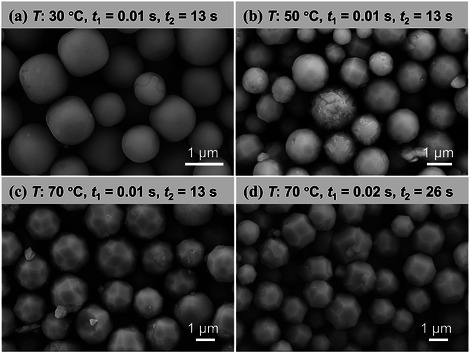
SEM images of Cu_2_O rounded cubes and polyhedrons with different facet numbers. Other synthesis conditions: After mixing: *C*_Cu_ = 0.003 m, *C*_AA_ = 0.03 m, *C*_NaOH_ = 0.85 m, *t*_3_ = 0 min; Flow rates: a–c) *F*_Cu_ = 36 mL min^−1^, *F*_NaOH_ = 12 mL min^−1^, *F*_AA_ = 6 mL min^−1^, d) *F*_Cu_ = 18 mL min^−1^, *F*_NaOH_ = 6 mL min^−1^, *F*_AA_ = 3 mL min^−1^.

To synthesize Cu_2_O polyhedrons, the NaOH and AA concentrations were significantly adjusted, and NaOH was added first, followed by AA. **Figure** **9**a–c show SEM images of Cu_2_O synthesized at different temperatures. At 30 °C, the relatively slow dissolution and regrowth rates promoted isotropic growth, resulting in rounded cubes with smooth surfaces. In contrast, at 70 °C, the markedly accelerated dissolution and regrowth induced anisotropic growth, yielding multi‐faceted Cu_2_O crystals, which suggests the possible coexistence of high‐index facets.^[^ ^18^, ^42^ ^]^ At higher temperatures, kinetic control may dominate over thermodynamic control. Increasing the residence time led to a decrease in the number of exposed facets (Figures 9c,d), indicating a transition from non‐equilibrium to thermodynamically favored equilibrium structures. Instead of using surface‐controlling agents, Cu_2_O polyhedrons with various facet configurations were effectively synthesized through kinetic control strategies. This approach eliminates the need for subsequent surfactant removal, providing clean Cu_2_O crystals for catalytic applications.

**Figure 10:**
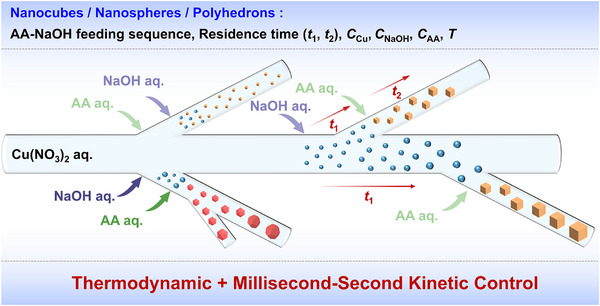
Schematic illustration of regulation strategies for morphology, size, and exposed facets in the continuous‐flow synthesis of Cu_2_O crystals.

Based on the above investigations, **Figure** **10** schematically illustrates the regulation strategies for the morphology, particle size, and exposed facet number of Cu_2_O crystals in the continuous‐flow synthesis system, highlighting the precise millisecond‐to‐second scale kinetic control approaches. Briefly, the morphology (cubes or polyhedrons) is predominantly governed by the reaction temperature and the concentrations of AA and NaOH. The number of exposed facets of the polyhedrons can be regulated by the residence time. The nanocube size is mainly affected by the AA‐NaOH feeding sequence, the residence time between AA and NaOH addition, as well as the concentrations of AA, NaOH, and the Cu precursor. Table S1 (Supporting Information) summarizes all the synthesis conditions and the effects of the synthesis parameters on morphology, size, and monodispersity.

## 4 Conclusion

In this study, a continuous‐flow synthesis system for Cu_2_O was developed to enable surfactant‐free, controllable, and scalable production. The design principles of the Cu_2_O flow synthesis system were first elucidated. The interval time between NaOH and AA addition significantly affects the Cu_2_O nanocube size. A shorter interval time (6→0.02 s) led to smaller Cu_2_O nanocubes (196→75 nm). The Reynolds number and particle growth time in the tubular reactor mainly influence nanocube uniformity, while aging time has a negligible effect. In addition, altering the feeding sequence of NaOH and AA (first AA then NaOH) drastically reduced the nanocube size from 76 to 14 nm with identical other synthesis conditions. Using the designed continuous‐flow system, uniform Cu_2_O nanocubes with sizes ranging from 14 to 200 nm were synthesized by adjusting the synthesis parameters. Moreover, non‐equilibrium Cu_2_O polyhedrons, likely featuring high‐index facets, were obtained under kinetically controlled conditions without the need for surfactant. Increasing residence time decreased the number of exposed facets, indicating a transition toward the thermodynamic equilibrium state. This millisecond‐to‐second scale kinetic control strategy enables precise control over nanocube size and capture of various facet configurations, which are difficult to achieve in batch processes. The continuous‐flow synthesis system also eliminates batch‐to‐batch variation and demonstrates clear potential for scale‐up, paving the way for the pragmatic applications of Cu_2_O nanocrystals.

## Conflict of Interest

The authors declare no conflict of interest.

## Author Contributions

The manuscript was collaboratively written with contributions from all authors. All authors have reviewed and approved the final version of the manuscript.

## Supporting information



Supporting Information

## Data Availability

The data that support the findings of this study are available in the supplementary material of this article.
