# Supplementary material for: Surfactant‐Free Continuous‐Flow Synthesis of Cu2O Crystals with Diverse Facets and Sizes
Source: Small Methods. 2025 Nov 29;10(1):e01927. doi: 10.1002/smtd.202501927 (PMC12790378; doi:10.1002/smtd.202501927)
Supplement: Supplementary file 1 — Supporting Information [file SMTD-10-e01927-s001.docx]

Supporting Information

Surfactant-free Continuous-flow Synthesis of Cu_2_O Crystals with Diverse Facets and Sizes

Chunli Han ^1^*, Akira Yoko ^1,2^*, Ardiansyah Taufik ^1^, Satoshi Ohara ^3^, and Tadafumi Adschiri ^1,3^*

^1^ WPI-Advanced Institute for Materials Research (WPI-AIMR), Tohoku University, Sendai 980-8577, Japan

^2^ International Center for Synchrotron Radiation Innovation Smart, Tohoku University, Sendai 980-8572, Japan

^3^ New Industry Creation Hatchery Center, Tohoku University, Sendai 980-8579, Japan

***** Corresponding author. E-mail: [tadafumi.ajiri.b1@tohoku.ac.jp](mailto:tadafumi.ajiri.b1@tohoku.ac.jp), [akira.yoko.c7@tohoku.ac.jp](mailto:akira.yoko.c7@tohoku.ac.jp), and [han.chunli.a6@tohoku.ac.jp](mailto:han.chunli.a6@tohoku.ac.jp)

**Contents**

**Fig. S1.** Effect of aging time on Cu_2_O crystals.

**Fig. S2.** SEM images of Cu_2_O synthesized at different AA concentrations.

**Fig. S3.** SEM images of Cu_2_O synthesized at different Cu precursor concentrations.

**Table S1.** Summary of synthesis conditions and the effects of synthesis parameters on particle size, morphology, and monodispersity.


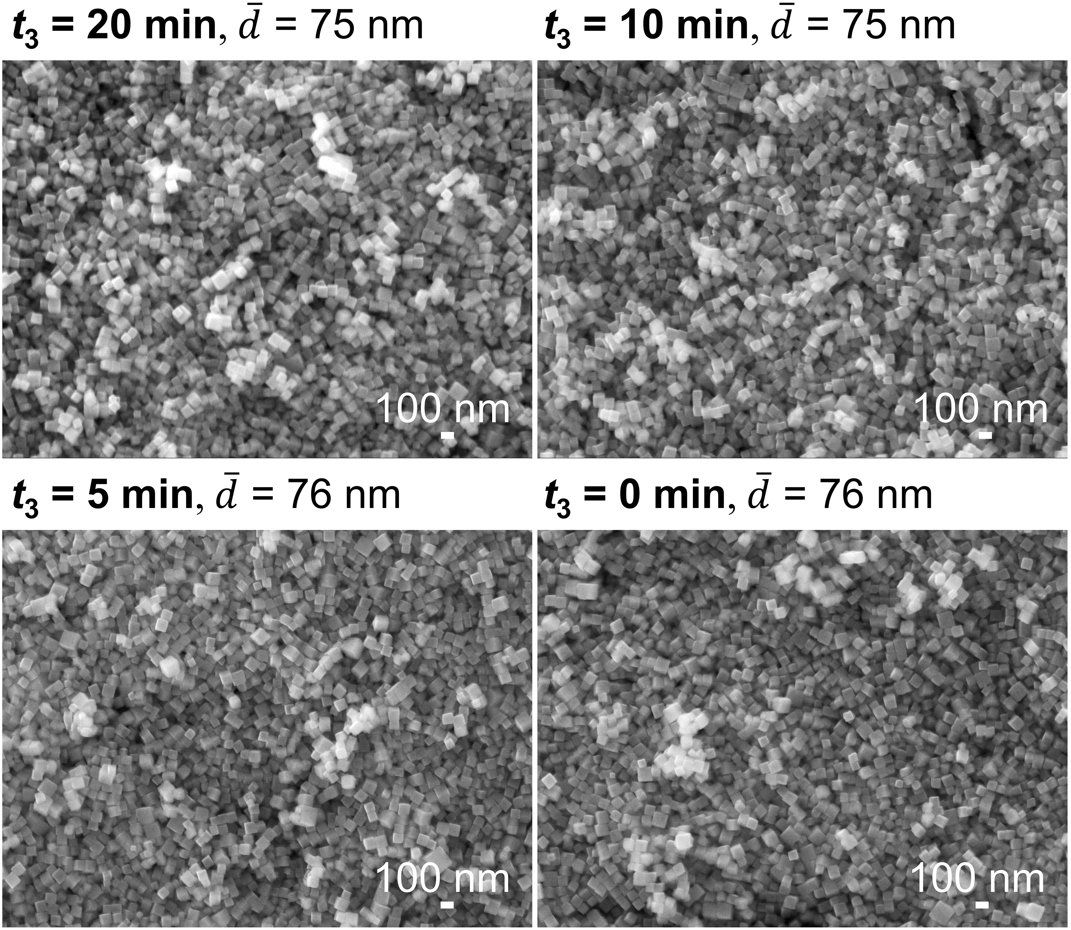


**Fig. S1.** Effect of aging time on Cu_2_O crystals. Other synthesis conditions: *T* = 30 ℃, After mixing: *C*_Cu_ = 0.002 M, *C*_AA_ = 0.01 M, *C*_NaOH_ = 0.02 M, *t*_1_ = 0.02 s, *t*_2_ = 26 s, sampling time: 1-2 min.


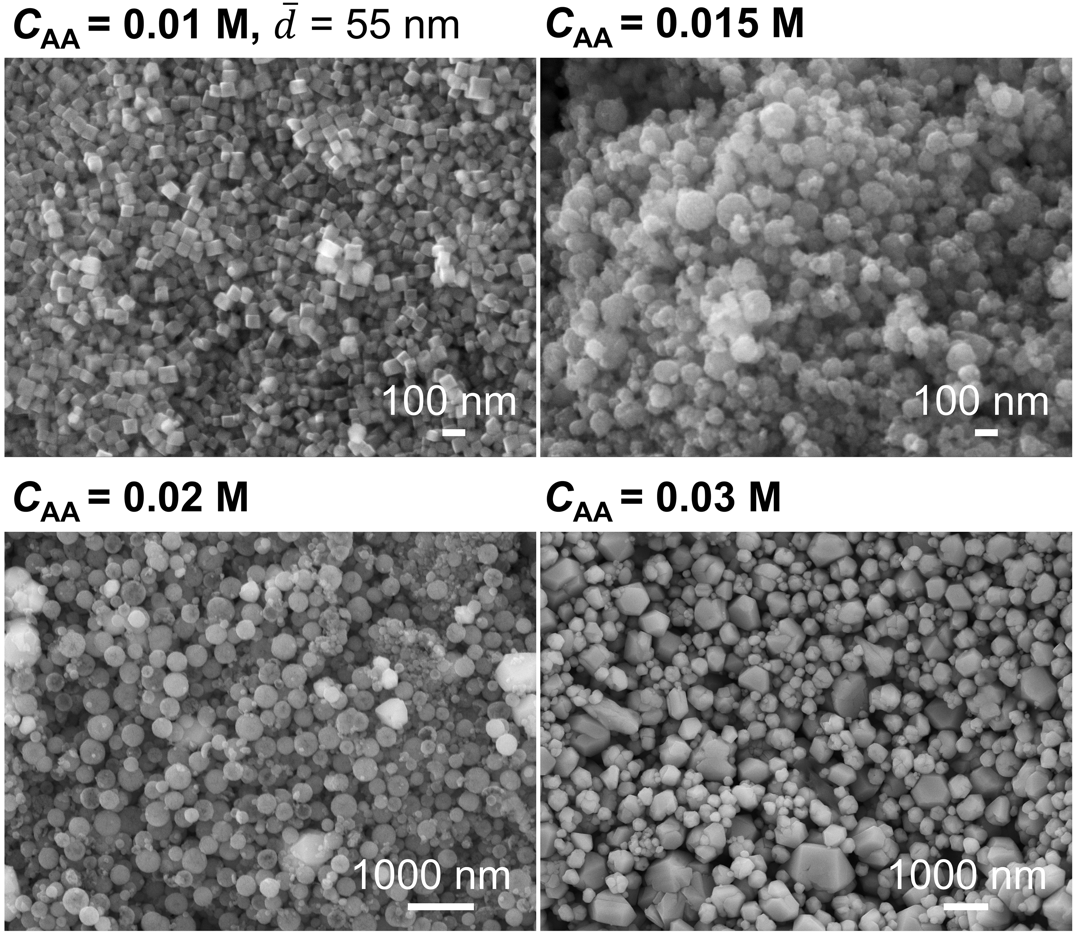


**Fig. S2.** SEM images of Cu_2_O synthesized at different AA concentrations. Other synthesis conditions: *T* = 30 ℃, After mixing: *C*_Cu_ = 0.002 M, *C*_NaOH_ = 0.015 M, *t*_1_ = 0.02 s, *t*_2_ = 26 s.


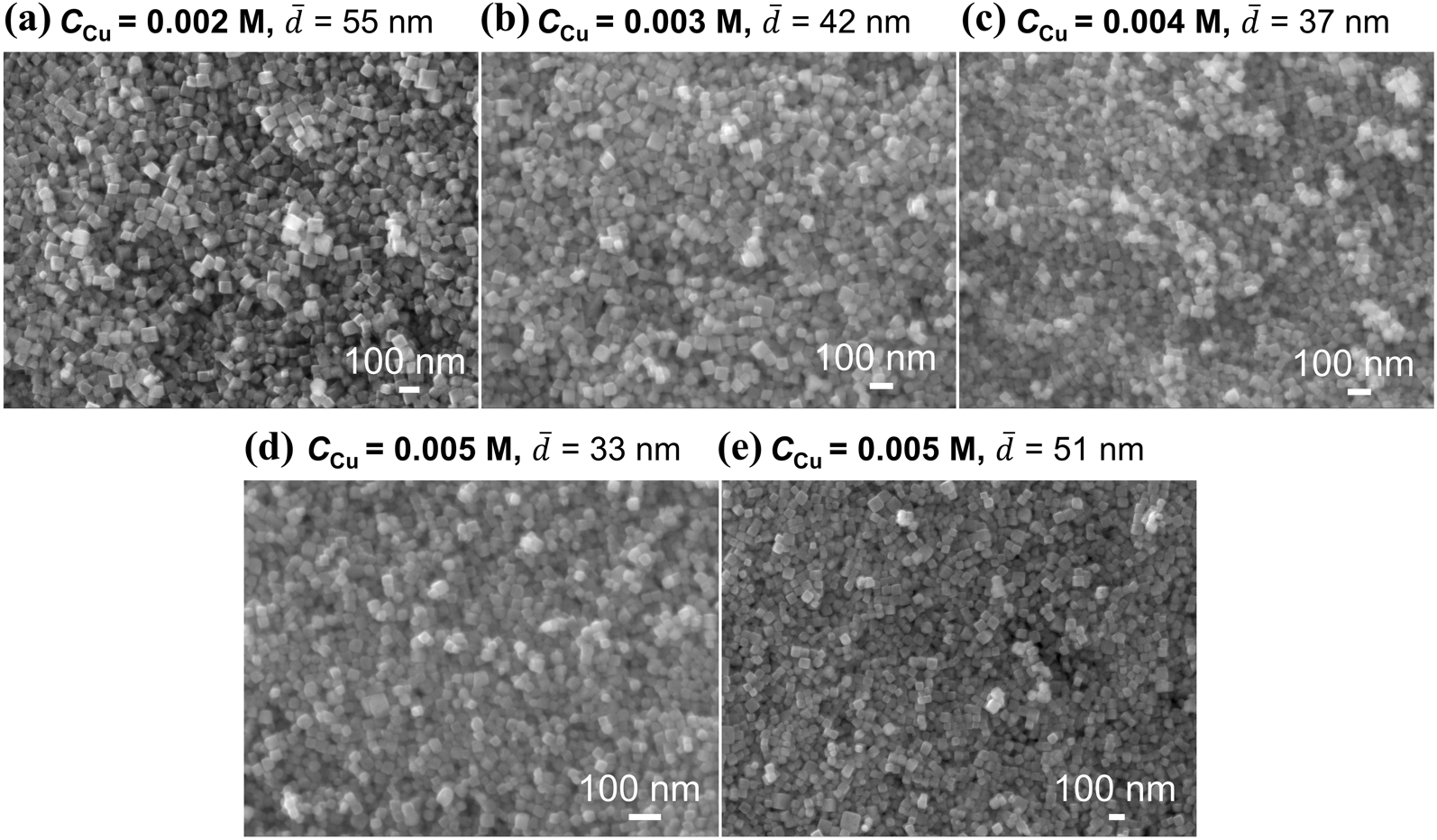


**Fig. S3.** (a-d) SEM images of Cu_2_O synthesized at different Cu precursor concentrations while keeping constant Cu(NO_3_)_2_:AA:NaOH molar ratio (2:10:15). Other synthesis conditions: *T* = 30 ℃, *t*_1_ = 0.02 s, *t*_2_ = 26 s. (e) SEM images of Cu_2_O synthesized at the following synthesis conditions: *T* = 30 ℃, After mixing: *C*_Cu_ = 0.005 M, *C*_NaOH_ = 0.037 M, *C*_AA_ = 0.0175 M, *t*_1_ = 0.02 s, *t*_2_ = 26 s. Compared with (d), the concentration of AA was slightly decreased.

**Table S1.** Summary of synthesis conditions and the effects of synthesis parameters on particle size, morphology, and monodispersity.

| **Sample** | ***C*_Cu_ /M** | ***C*_AA_ /M** | ***C*_NaOH_ /M** | ***t*_1_ /s** | ***t*_2_ /s** | ***t*_3_ /min** | ***T* /℃** | $\bar{\boldsymbol{d}}$ **/nm** | **Morphology** | **Monodispersity** |
| --- | --- | --- | --- | --- | --- | --- | --- | --- | --- | --- |
| 1 ^a^ | 0.002 | 0.01 | 0.020 | 0.02 | 26 | 0 | 30 | 14 | Nanocube | Uniform |
| 2 | 0.002 | 0.01 | 0.020 | 0.02 | 26 | 20 | 30 | 75 | Nanocube | Uniform |
| 3 | 0.002 | 0.01 | 0.020 | 0.15 | 26 | 20 | 30 | 129 | Nanocube | Uniform |
| 4 | 0.002 | 0.01 | 0.020 | 0.48 | 26 | 20 | 30 | 142 | Nanocube | Uniform |
| 5 | 0.002 | 0.01 | 0.020 | 6.03 | 26 | 20 | 30 | 196 | Nanocube | Uniform |
| 6 | 0.002 | 0.01 | 0.020 | 0.02 | 6 | 0 | 30 | 71 | Nanocube | Un-uniform |
| 7 | 0.002 | 0.01 | 0.020 | 0.02 | 26 | 0 | 30 | 76 | Nanocube | Uniform |
| 8 | 0.002 | 0.01 | 0.020 | 0.02 | 26 | 5 | 30 | 76 | Nanocube | Uniform |
| 9 | 0.002 | 0.01 | 0.020 | 0.02 | 26 | 10 | 30 | 75 | Nanocube | Uniform |
| 10 | 0.002 | 0.01 | 0.020 | 0.02 | 26 | 20 | 30 | 75 | Nanocube | Uniform |
| 11 | 0.001 | 0.01 | 0.015 | 0.02 | 26 | 20 | 30 | 71 | Nanocube | Uniform |
| 12 | 0.002 | 0.01 | 0.015 | 0.02 | 26 | 20 | 30 | 55 | Nanocube | Uniform |
| 13 | 0.003 | 0.01 | 0.015 | 0.02 | 26 | 20 | 30 | 25 | Nanocube | Medium |
| 14 | 0.002 | 0.01 | 0.015 | 0.02 | 26 | 20 | 30 | 55 | Nanocube | Uniform |
| 15 | 0.002 | 0.015 | 0.015 | 0.02 | 26 | 20 | 30 | 50-200 | Porous nanospheres | Un-uniform |
| 16 | 0.002 | 0.02 | 0.015 | 0.02 | 26 | 20 | 30 | 100-400 | Smooth nanospheres | Un-uniform |
| 17 | 0.002 | 0.03 | 0.015 | 0.02 | 26 | 20 | 30 | — | Irregular particles | Un-uniform |
| 18 | 0.002 | 0.01 | 0.020 | 0.02 | 26 | 20 | 30 | 75 | Nanocube | Uniform |
| 19 | 0.002 | 0.01 | 0.015 | 0.02 | 26 | 20 | 30 | 55 | Nanocube | Uniform |
| 20 | 0.002 | 0.01 | 0.010 | 0.02 | 26 | 20 | 30 | 159 | NanoSpheres | Un-uniform |
| 21 | 0.002 | 0.01 | 0.015 | 0.02 | 26 | 20 | 10 | 54 | Nanocube | Uniform |
| 22 | 0.002 | 0.01 | 0.015 | 0.02 | 26 | 20 | 20 | 51 | Nanocube | Uniform |
| 23 | 0.002 | 0.01 | 0.015 | 0.02 | 26 | 20 | 30 | 55 | Nanocube | Uniform |
| 24 | 0.002 | 0.01 | 0.015 | 0.02 | 26 | 20 | 50 | — | Very small particles | Un-uniform |
| 25 | 0.003 | 0.03 | 0.849 | 0.01 | 13 | 0 | 30 | 1000-2000 | Rounded cubes | Un-uniform |
| 26 | 0.003 | 0.03 | 0.849 | 0.01 | 13 | 0 | 50 | 1000-2000 | Polyhedrons | Un-uniform |
| 27 | 0.003 | 0.03 | 0.849 | 0.01 | 13 | 0 | 70 | 1000-2000 | Polyhedrons | Un-uniform |
| 28 | 0.003 | 0.03 | 0.849 | 0.02 | 26 | 0 | 70 | 1000-2000 | Polyhedrons | Un-uniform |

1. For sample 1, the reductant AA was introduced first, followed by NaOH. For the other samples, NaOH was added first, followed by AA.
